# In Silico–Based Investigation of the Immunogenicity and Biochemical Attributes of *Toxoplasma gondii* Apical Membrane Antigen 1 (TgAMA1)

**DOI:** 10.1155/japr/3514414

**Published:** 2025-04-12

**Authors:** Masoud Foroutan, Ali Dalir Ghaffari, Fatemeh Ghaffarifar, Amir Karimipour-Saryazdi, Arezo Arzani Birgani, Hamidreza Majidiani, Hélder Cortes, Hany M. Elsheikha

**Affiliations:** ^1^Department of Basic Medical Sciences, Faculty of Medicine, Abadan University of Medical Sciences, Abadan, Iran; ^2^Department of Parasitology and Mycology, Faculty of Medicine, Shahed University, Tehran, Iran; ^3^Department of Parasitology, Faculty of Medical Sciences, Tarbiat Modares University, Tehran, Iran; ^4^Department of Health Information Technology, Abadan University of Medical Sciences, Abadan, Iran; ^5^Healthy Aging Research Centre, Neyshabur University of Medical Sciences, Neyshabur, Iran; ^6^Department of Basic Medical Sciences, Neyshabur University of Medical Sciences, Neyshabur, Iran; ^7^Laboratório de Parasitologia Victor Caeiro, MED (Mediteranean Institute for Agriculture, Environment and Development), University of Evora, Évora, Portugal; ^8^School of Veterinary Medicine and Science, Faculty of Medicine and Health Sciences, University of Nottingham, Loughborough, UK

**Keywords:** apical membrane antigen 1, bioinformatics, in silico, *Toxoplasma gondii*

## Abstract

**Background:** Apical membrane antigen 1 (AMA1) is a highly conserved microneme protein in apicomplexan parasites. In this study, immunoinformatics tools and in silico protein structure prediction were used to characterize the structure, physicochemical properties, posttranslational modification sites, immunogenic epitopes, allergenicity, and immune simulation of the *Toxoplasma gondii* AMA1 (TgAMA1) protein.

**Methods:** A comprehensive analysis was performed using multiple bioinformatics web servers to analyze the antigenicity, physicochemical features, secondary and tertiary structures, B and T cell epitopes, and in silico immune simulation of TgAMA1.

**Results:** The analysis revealed that the AMA1 protein consists of 569 amino acid residues and has a molecular weight of approximately 63 kDa. The grand average of hydropathicity (GRAVY) was -0.531 and the aliphatic index was calculated as 64.62. Based on the GOR IV server, TgAMA1 contained 20.21% alpha helices, 58.52% random coils, and 21.27% extended strands. The Ramachandran plot of the refined model revealed that over 97% of the residues were located in the favored region. The AMA1 protein was highly immunogenic and nonallergenic in nature. In silico immune simulation using the C-ImmSim server suggested that three doses of TgAMA1 would elicit potent humoral and cell-mediated immune responses.

**Conclusion:** These findings provide valuable insights for further in vitro and in vivo investigations of TgAMA1's potential as a vaccine candidate against toxoplasmosis.

## 1. Introduction

*Toxoplasma gondii*, a protozoan parasite, is one of the most prevalent pathogens in the phylum Apicomplexa, affecting both humans and animals [1]. The *T. gondii* parasite poses significant socioeconomic and health risks [2–4]. It is also an important abortifacient agent for farm animals such as sheep, goats, and pigs [5]. The definitive hosts for *T. gondii* are exclusively felids, while humans and other warm-blooded species serve as intermediate hosts [1, 6]. In healthy individuals with a competent immune system, *T. gondii* infection typically presents with mild or no symptoms; however, it can result in serious health consequences in fetuses infected in utero [7] or in immunosuppressed individuals, such as those living with acquired immunodeficiency syndrome (AIDS) [4, 8].

The parasite's life cycle consists of three forms: sporozoites in oocysts, bradyzoites in tissue cysts, and tachyzoites. Bradyzoites are associated with the chronic stage of infection, whereas tachyzoites are involved in the acute phase [9] resulting into encephalitis, myocarditis, ocular and neurological impairment, and systemic complications [4]. Currently, the only effective treatment for *T. gondii* infection targets tachyzoites; however, it cannot eliminate the parasite's tissue cysts [10]. Therefore, the development of a vaccine is critical for managing infection in both domestic animals and humans, considering the significant global burden of toxoplasmosis [11–13]. Vaccination is broadly regarded as the most effective strategy for controlling infectious diseases by providing long-lasting immunity [14, 15]. Several approaches have been explored, including live attenuated, inactivated, subunit, protein or DNA, and vector-based live vaccines, to develop vaccines, to control toxoplasmosis [11, 12, 16–19].

Studies have shown that vaccines containing immunogenic components of antigenic molecules can stimulate both humoral and cellular immunity, providing durable protection against infection [19, 20]. Identifying and isolating efficient epitopes that activate the immune system is a crucial step in developing epitope-based vaccines [21]. Immunoinformatics can be used to identify the potential B and T cell epitopes of antigenic molecules, enabling the design of multiple epitope subunit vaccine that elicit strong humoral and cellular responses [22].

Several molecular components expressed during various stages of *T. gondii's* life cycle have been identified as potential vaccine candidates, including surface antigens (SAGs) and secretory proteins such as microneme proteins (MICs), rhoptry proteins (ROPs), and dense granule antigens (GRAs) [12, 16, 17, 23]. However, no commercially licensed vaccine for human use is available yet. An ideal *T. gondii* vaccine should enhance CD8^+^ and CD4^+^ T cell–mediated cellular responses and increase the production of proinflammatory cytokines such as interferon-gamma (IFN-*γ*) [24, 25].

Apical membrane antigen 1 (AMA1), a highly conserved MIC in apicomplexan parasites, has garnered considerable attention as a potential malaria vaccine candidate [26]. AMA1 facilitates host cell recognition and parasite entry by forming a moving junction complex. It has demonstrated adequate immunogenicity and protection against acute and chronic toxoplasmosis in vivo [26–29]. Immunological and genetic studies have further confirmed AMA1's role in parasite invasion and pathogenesis [26, 30–33], highlighting its promising potential for vaccine development. This study is aimed at investigating the physicochemical and structural properties, immunogenic epitopes, and other useful features of the TgAMA1 protein using a range of bioinformatics tools.

## 2. Materials and Methods

### 2.1. TgAMA1 Protein Sequence Retrieval

The protein sequence of TgAMA1 protein (ToxoDB Number TGME49_255260) was retrieved from the ToxoDB web server [34] and stored in FASTA format for subsequent analyses. The links to all bioinformatics servers employed in this study are provided in Table 1.

### 2.2. Allergenicity, Antigenicity, Solubility, and Physicochemical Characteristics

To predict antigenicity, VaxiJen v2.0 [69] was used. This program transforms protein sequences using auto and cross variance (ACC) transformation, generating uniform vectors based on the primary physicochemical properties of amino acids. By focusing on these characteristics, VaxiJen predicts protein antigenicity without requiring sequence alignment. For allergenicity assessment, AllergenFP 1.0 and AlgPred were used to identify IgE-specific epitopes and allergen motifs within the submitted sequences. These servers use the Multiple EM for Motif Elicitation (MEME)/Motif Alignment and Search Tool (MAST) approach to predict potential allergenic properties [36, 37]. Additionally, protein solubility was evaluated using the Protein-Sol web server. Predictions were based on solubility scores, with values exceeding a predefined threshold considered soluble according to the experimental dataset's population average [61].

The ExPASy ProtParam server [40] was used to determine the physicochemical properties of TgAMA1, including molecular weight (MW), half-life, isoelectric point (pI), aliphatic and instability indices, and grand average hydrophilicity (GRAVY). The instability index indicates protein stability, whereas the aliphatic index measures thermotolerance based on aliphatic side chain mass. The pI corresponds to the pH at which a protein has no net charge, and GRAVY values reflect the protein's hydropathicity [40, 70, 71].

### 2.3. Evaluation of the Subcellular Location and Transmembrane Domains of the AMA1

We employed the TMHMM ver. 2.0 and PSORT II prediction servers to predict the subcellular location and transmembrane structure of the AMA1 protein, respectively [62, 67, 68].

### 2.4. Posttranslational Modification (PTM) Site Prediction

The PTM sites of the TgAMA1 protein, including N-glycosylation, O-glycosylation, phosphorylation, and acetylation sites, were predicted using the web servers NetNGlyc 1.0 [56], NetOGlyc 4.0 [57], NetPhos 3.1 [58], and GPS-PAIL 2.0 [44], respectively. Except for NetNGlyc and acetylation, which were dependent on “all Asn residues” and “all types,” respectively, the parameter of prediction for the aforementioned databases was set to default.

### 2.5. Secondary and Three-Dimensional (3D) Structures of the TgAMA1

The TgAMA1 protein was subjected to secondary structure component analysis using the Garnier–Osguthorpe–Robson (GOR) IV server, with a prediction accuracy of 64.6% [43]. In addition, the online tool SWISS-MODEL was used to model the 3D homology of the protein using the default parameters [66].

### 2.6. Refinement of the 3D Model and Subsequent Validation

The 3D model was refined using the GalaxyRefine server [41, 42]. This server performs side chain repacking and rebuilding first, followed by molecular dynamic simulation for overall structure relaxation. The model was further validated using the SWISS-MODEL structure assessment service. The Ramachandran plot was employed to analyze the preferred dihedral angles of the protein backbone [64], and ProSA-web was used to assess the model's overall quality [60].

### 2.7. Prediction of Epitopes for Cytotoxic T Lymphocytes (CTLs)

The NetCTL 1.2 (with a threshold of 0.75) was applied to predict the four highest-ranking epitopes of CTL. Three supertypes (A3, A2, and B7) were applied to cover 88.3% of the global population [55, 72]. The predicted epitopes were evaluated for immunogenicity using the MHC Class I immunogenicity prediction server in the Immune Epitope Database (IEDB) [51].

### 2.8. Prediction of Helper T Lymphocyte (HTL) Epitope and Screening for IL-4 and IFN-*γ* Induction and Antigenicity

The complete set of human leukocyte antigens (HLAs) was used to predict HTL epitopes using the IEDB MHCII binding prediction tool. The highest affinity is for each unique HLA allele, which has the lowest percentile rank among all epitopes [52]. The IL4-pred [54], IFNepitope [53], and VaxiJen v2.0 [69] web servers were used to predict the antigenicity and possibility of inducing IFN-*γ* and IL-4 for every epitope.

### 2.9. Prediction of Continuous B Cell Epitopes in Multiple Steps by Screening for Allergenicity, Water Solubility, and Antigenicity

Two web servers, ABCpred [35] and SVMTriP [65], were employed to forecast each protein's linear B cell epitopes. The ABCpred server uses a recurrent neural network (RNN) algorithm to predict B cell epitopes with 65.93% accuracy (threshold set to 0.9) [35]. The SVMTriP server provides improved prediction performance by integrating a support vector machine (SVM) with tripeptide similarity and propensity scores [65]. Common linear B cell epitopes were chosen and evaluated in terms of antigenicity, allergenicity, and water solubility using the internet tools VaxiJen v2.0, AllergenFP v1.0, and PepCalc, respectively [37, 59, 69]. Both protein stability and solubility are crucial biophysical factors in the rational design of multiepitope vaccines. Any values > 0.45 are considered soluble [61]. Furthermore, when the IEDB server was used, additional factors, such as surface accessibility, flexibility, beta-turns, antigenicity, and hydrophilicity, were predicted [45–50]. Moreover, with the Protein Data Bank (PDB) file, ElliPro was utilized to predict discontinuous B cell epitopes [39].

### 2.10. In Silico Immune Simulation

Using the C-ImmSim server to simulate immune responses, the effectiveness of TgAMA1 was tested. This server computes the immune system's reaction to an antigen using a position-specific scoring matrix (PSSM). Along with identifying immune interactions and immune epitopes, the server also analyzes the humoral and cellular responses of the mammalian immune system to a given antigen [38]. The following parameters were set for in silico immune simulation by this web-based server: three injections of TgAMA1 at 4-week intervals, with time series of 1, 84, and 168 (each time step is considered 8 h in real life), a random seed of 12345, a simulation volume of 10, and 1050 simulation steps.

## 3. Results

### 3.1. Physicochemical and Immunological Characteristics of the TgAMA1 Protein

In terms of antigenicity, the TgAMA1 protein exhibited good antigenicity (0.6685). The AlgPred server analysis revealed that the TgAMA1 sequences lacked features corresponding to specific IgE epitopes and MEME/MAST motifs. Furthermore, AllergenFP 1.0 was utilized, and the results showed that this protein is not allergenic. The predicted scaled solubility by the Protein-Sol server was estimated to be 0.317. The MW of TgAMA1 was 63 kDa. Based on its estimated pI of 5.78, the TgAMA1 protein was relatively acidic. The estimated half-life (mammalian reticulocytes, in vitro) of the protein was 30 h. TgAMA1 was unstable based on the stability threshold of < 40 for the instability index. The aliphatic index of the TgAMA1 protein was high. The negative GRAVY score of the TgAMA1 protein indicated that it has a hydrophilic nature.  Table 2 shows the results of the physicochemical characterization.

### 3.2. Prediction of the Subcellular Location and Transmembrane Domains of the TgAMA1 Protein

**Figure 1:**
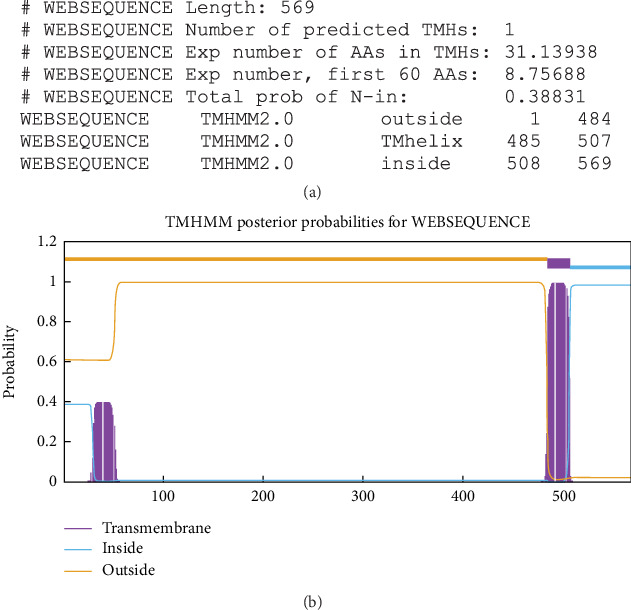
Prediction of AMA1 protein transmembrane helices. (a) A list of the anticipated locations of the intervening loop regions and transmembrane helices, along with some statistics, is shown. (b) Investigation of the TgAMA1 transmembrane domain.

As shown in Figure 1, the TMHMM server results indicate that the AMA1 sequence consists of a single transmembrane domain. Using PSORT II, the subcellular localization of AMA1 was predicted to be as follows: 4.3% cytoplasmic, 30.4% plasma membrane, 4.3% cytoskeletal, 13.0% mitochondrial, 4.3% peroxisomal, and 4.3% extracellular, including the cell wall, 4.3% Golgi, 17.4% nuclear, and 17.4% endoplasmic reticulum.

### 3.3. Prediction of PTM Locations

**Figure 2:**
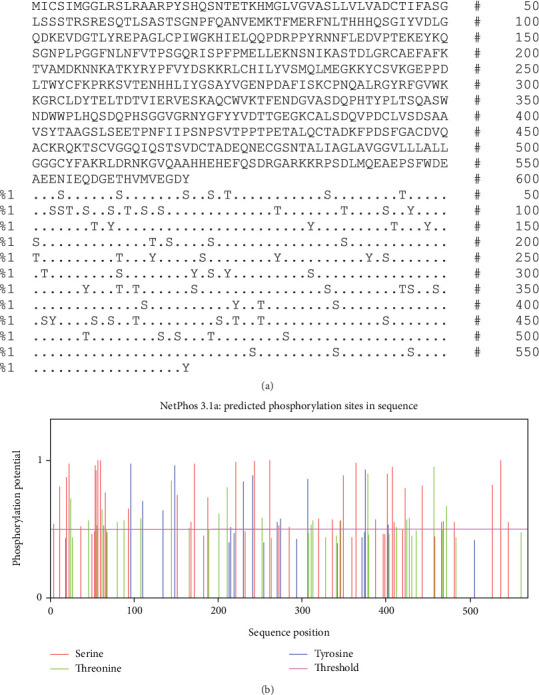
Bioinformatics analysis of the phosphorylation sites of the AMA1 sequence. (a) The number of predicted sites, based on S (serine), T (threonine), and Y (tyrosine). (b) Prediction diagram of TgAMA1 phosphorylation sites.

The AMA1 sequence contains two N-glycosylation sites. O-glycosylation regions were abundant in AMA1 (23 sites). The NetPhos 3.1 and GPS-PAIL 2.0 servers were used to examine the phosphorylation and acylation sites of the TgAMA1 protein. The analysis revealed 72 phosphorylation sites (Ser: 38, Thr: 21, and Tyr: 13) (Figure 2a,b). Additionally, one acetylation region was found in TgAMA1.

### 3.4. Prediction of Secondary and 3D Structures

**Figure 3:**
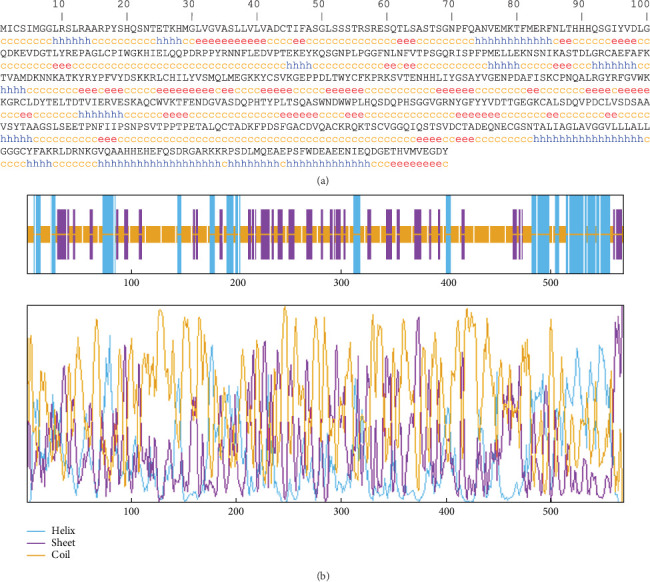
(a) Predicted secondary structure of the TgAMA1 sequence by the GOR IV online server. h = helix, e = extended strand, and c = coil. (b) Graphical outputs for secondary structure prediction of TgAMA1 provided by GOR IV.

**Figure 4:**
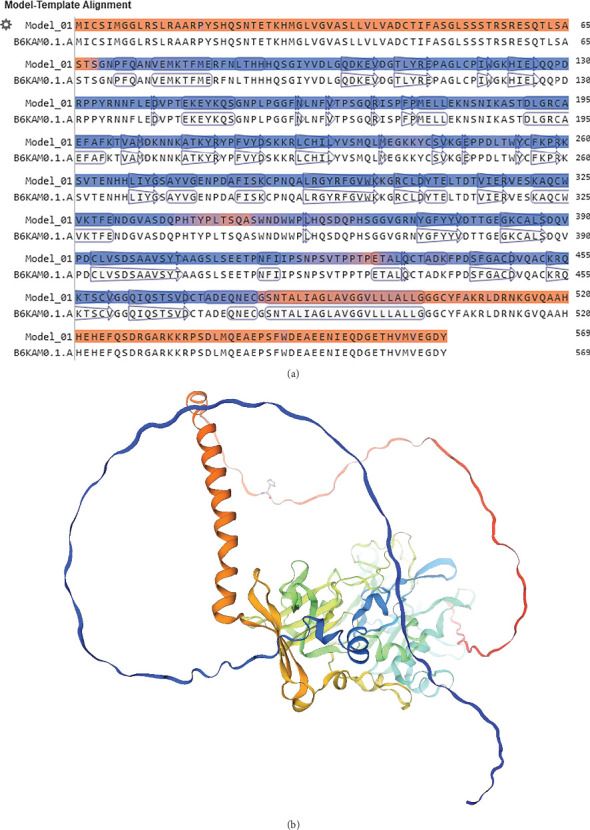
SWISS-MODEL server output. (a) Model-template alignment. (b) Three-dimensional structure of the TgAMA1 protein.

The most common component of the secondary structure in the AMA1 sequences, according to the prediction of the secondary structure via the GOR IV server, was the random coil, accounting for 58.52% of the secondary structure, with the extended strands and alpha helices representing 21.27% and 20.21%, respectively (Figure 3a,b). The AMA1 protein sequence homology modeling results were obtained using the SWISS-MODEL web server. For monomer template 1 of the TgAMA1 protein, a coverage of 1.00 was determined, indicating 100% sequence identity (Figure 4).

### 3.5. 3D Model Validation and Refinement

**Figure 5:**
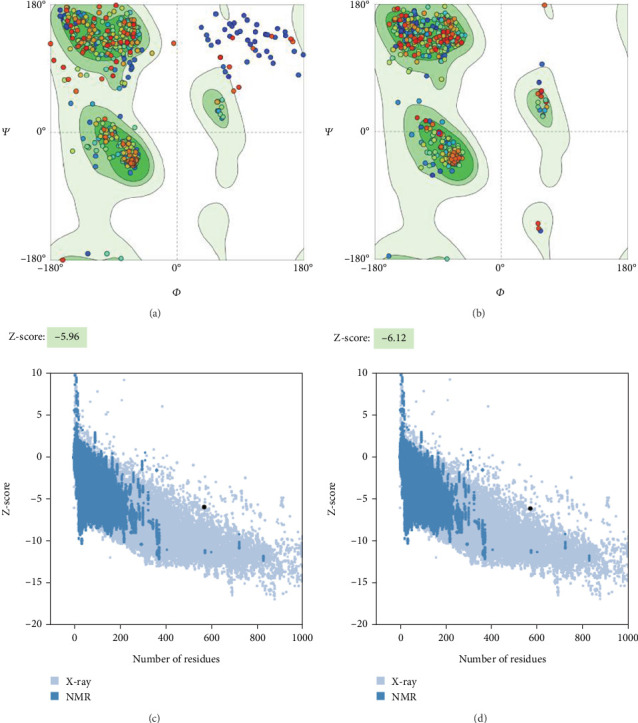
Validation of the 3D model of the TgAMA1 protein using a Ramachandran plot and ProSA-web. (a, c) Crude models. (b, d) Refined models.

The 3D structure was refined using the GalaxyRefine program. Following refinement, an improvement in the quality of the 3D structure was noted based on the results of the Ramachandran plot and ProSA-web online tools. Before the protein was refined, its immunogenic efficiency was confirmed by the structure assessment service of the SWISS-MODEL tool, which validated the protein and revealed that 86.07% of the residues were incorporated in the favored regions (Figure 5). Following refinement, Ramachandran plots were generated, and 97.88% of the residues were located in the favored regions (Figure 5). A measure of the model's quality is the *Z* score; in the original model (based on the ProSA-web), this parameter was −5.96, and most residues were located in the favored regions (Figure 5). The *Z* score also suggested that the quality of the 3D structure improved after refinement (−6.12) (Figure 5).

### 3.6. Prediction and Screening of the HTL and CTL Epitopes

The NetCTL 1.2 server and additional immunogenicity screening revealed that the four most immunogenic epitopes among the MHC I supertypes A2, A3, and B7 were RGYRFGVWK (score: 1.2287, immunogenicity: 0.3610), LVADCTIFA (score: 1.0980, immunogenicity: 0.1997), and TPPTPETAL (score: 1.2916, immunogenicity: 0.1745), respectively. Table S1 presents the comprehensive outcomes of CTL epitope prediction and screening.

HTL epitope prediction was limited to the entire IEDB server HLA reference set. Five top-ranked epitopes were chosen for the protein under investigation. Among all the predicted epitopes, the most antigenic peptide was AVSYTAAGSLSEETP (VaxiJen score: 1.0580), followed by FKTVAMDKNNKATKY (VaxiJen score: 0.8144) and GKHIELQPDRPPYR (VaxiJen score: 0.6116). The three AMA1 epitopes exhibited strong IFN-*γ*-inducing properties. Likewise, all of the epitopes were potent IL-4 inducers (Table S2).

### 3.7. Prediction and Screening of Continuous B Cell Epitopes

**Figure 6:**
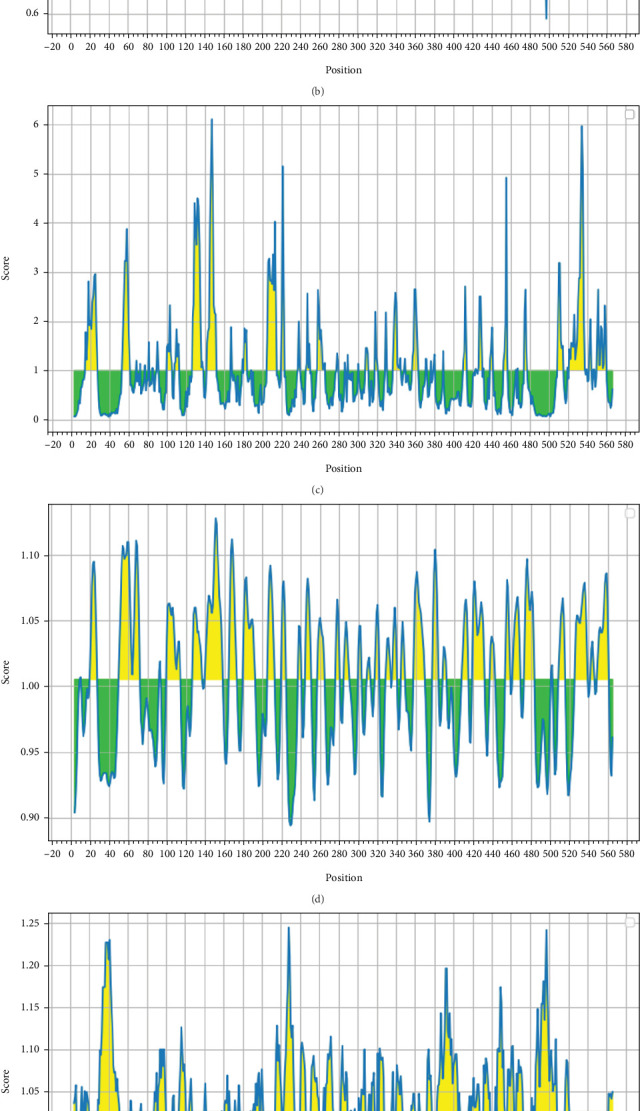
The output of the Immune Epitope Database (IEDB) was obtained from the online server. (a) BepiPred linear epitope prediction 2.0. (b) Beta-turn. (c) Surface accessibility. (d) Flexibility. (e) Antigenicity. (f) Hydrophilicity. The *x*-axes represent the residue positions in the sequence, while the *y*-axes represent the corresponding score for each residue; yellow color represents that the residue might have a greater probability of being a part of the epitope, and green color represents the unfavorable regions relevant to the properties of interest.

**Figure 7:**
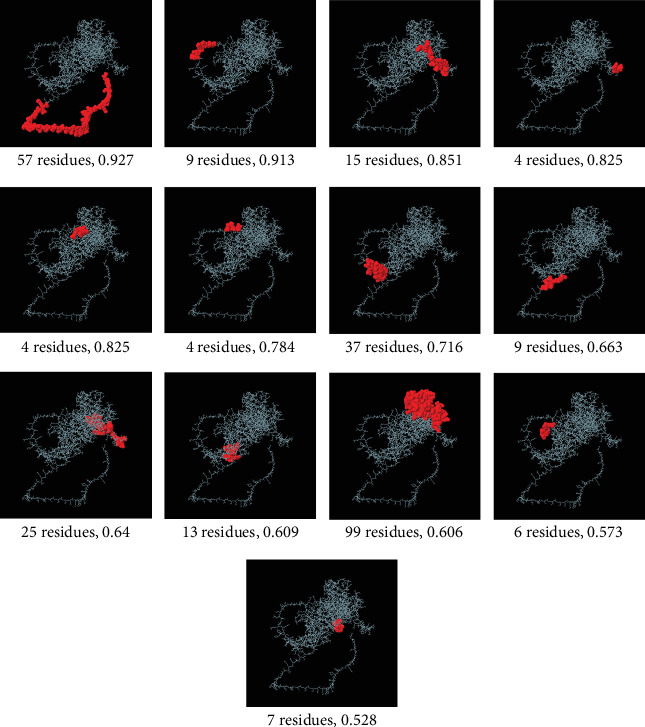
Discontinuous 3D structure of B cell epitopes on the TgAMA1 protein predicted by ElliPro. The lengths of the epitopes and their scores are indicated. The white rods and red domains indicate the TgAMA1 protein and conformational B cell epitopes, respectively.

After using two different servers for predicting continuous B cell epitopes (SVMTriP and ABCpred) to confirm the results, the epitopes were further studied to assess their antigenicity, allergenicity, and solubility in water. The most antigenic, nonallergic B cell epitopes for the AMA1 protein that had good water solubility were “CSVKGEPPDLTWYCFK” from ABCpred (VaxiJen score: 1.1396) and “ELLEKNSNIKASTDLG” from SVMTriP (VaxiJen score: 0.9435) (Table S3). According to the IEDB prediction, the average score predicted for the TgAMA1 protein was as follows: BepiPred linear epitope prediction, 0.51; antigenicity, 1.025; beta-turn, 1.027; hydrophilicity, 2.086; surface accessibility, 1.000; and flexibility, 1.005. The outputs of the IEDB server are shown in Figures 6a, 6b, 6c, 6d, 6e, and 6f. Finally, the use of the ElliPro tool in the IEDB server predicted 13 discontinuous B cell epitopes (Figure 7 and Table S4).

### 3.8. Immune System Simulation Against TgAMA1

**Figure 8:**
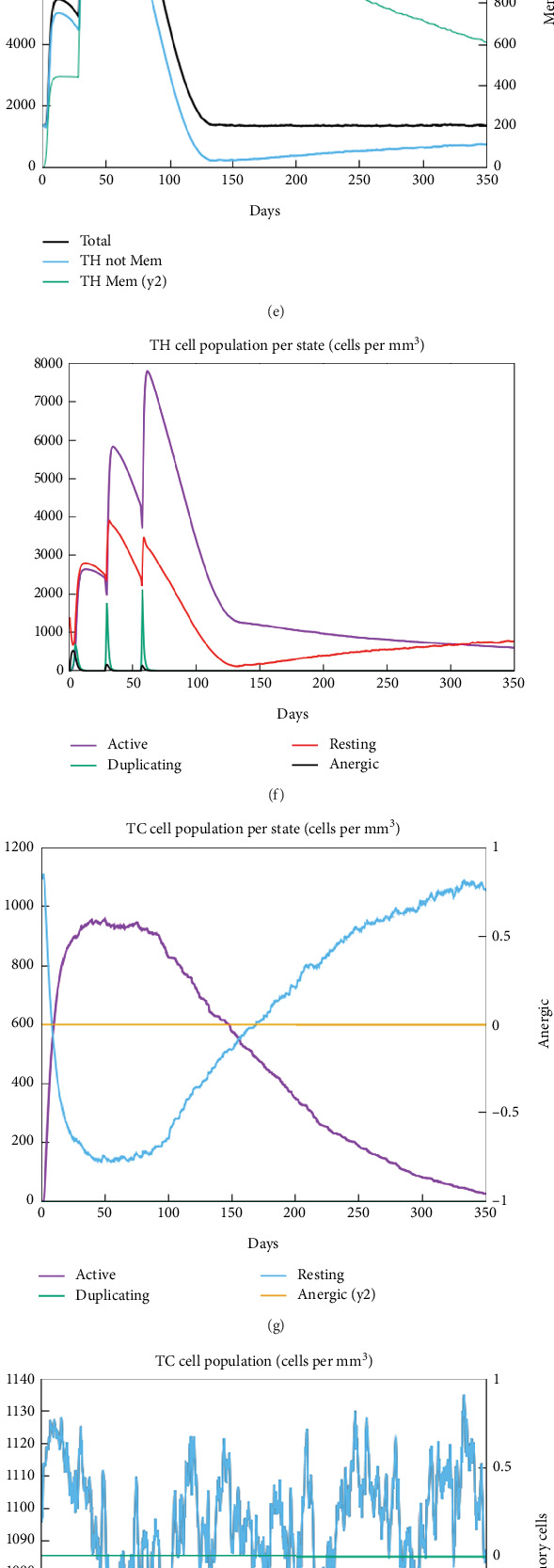
The results of the C-ImmSim server revealed the immune profile. (a) Immunoglobulin production. (b, c) B cell population (cells per cubic millimeter). (d) Plasma B lymphocyte count subdivided per isotype (IgM, IgG1, and IgG2). (e, f) T-CD4^+^ cell population (cells per cubic millimeter). (g, h) T-CD8^+^ cell population (cells per cubic millimeter). (i) Dendritic cells per state (cells per cubic millimeter). (j) NK cell population (cells per cubic millimeter). (k) Macrophages. Total count, internalized, presenting on MHC Class II, active and resting macrophages (cells per cubic millimeter). (l) Level of cytokine production (nanogram/milliliter) by TgAMA1.

With every injection and subsequent exposure to the TgAMA1 protein, the antibody response markedly increased. Increased IgM antibody levels indicate a dominant immune response. As shown in Figure 8, upon TgAMA1 exposure, high titers of IgM, IgG_1_, and a combination of both [IgG + IgM] were predicted. Additionally, increased B cell density was linked to increased secretion of IgG_1_ + IgG_2_, IgM, and IgG + IgM antibodies. A considerable increase in memory B cell counts and a drop in antigen concentrations subsequently occurred (Figures 8, 8, and 8). Both T-CD4^+^ and T-CD8^+^ cells started to duplicate a few days after TgAMA1 injection and were present for up to 350 days (Figures 8, 8, 8, and 8). Additionally, IFN-*γ* and IL-2 levels increased after immunization (Figure 8). These findings suggest that the administration of TgAMA1 as a vaccine candidate could trigger significant immune responses (Figures 8, 8, 8, 8, 8, 8, 8, 8, 8, 8, 8, and 8).

## 4. Discussion

Developing vaccines against parasitic infections remains challenging due to the complexity of the life cycles and their poorly understood immune evasion mechanisms [73]. The significant public health and veterinary burden of *T. gondii* infection [2, 3, 6, 74, 75], combined with the limitations of current treatments [76], underscores the urgent need for more effective control strategies, such as vaccines [13, 76]. Despite significant efforts [19, 20], no vaccine has yet been able to completely eradicate tissue cysts or prevent congenital transmission [16, 77]. Advancements in genomics, sequencing technologies, and computational biology have facilitated the development of immunoinformatics tools [22, 78, 79], which enable researchers to predict the biochemical properties of proteins in silico and identify the most immunogenic B cell and T cell epitopes, supporting the rational design of multiepitope vaccines [63, 80–82].

The survival of *T. gondii* depends on its ability to invade and colonize host cells, a process mediated by proteins, such as AMA1. This highly conserved microneme-secreted protein plays a crucial role in the formation of tight junctions between the parasite and host cell membrane [26, 29]. However, little is known about the immunogenic epitopes and physicochemical properties of TgAMA1. To address this gap, this study was carried out to provide new insight into its immunogenicity and functional properties.

Using the ToxoDB web server, the amino acid sequence of *T. gondii* AMA1 was retrieved and antigenicity was predicted using VaxiJen v2.0. Allergenicity was assessed using AllergenFP 1.0 and AlgPred, confirming that AMA1 is immunogenic (0.6685) and nonallergenic. Given that an ideal vaccine candidate must lack allergenicity [83], this characteristic strengthens the potential suitability of the AMA1 protein. Physicochemical analysis further revealed that AMA1 is hydrophilic and thermostable over a wide temperature range and possesses a suitable molecular weight and a half-life of 30 h, facilitating its experimental validation and purification [84]. Further investigation of PTMs identified two N-glycosylation sites, 23 O-glycosylation sites, 72 phosphorylation sites, and a single acetylation region, indicating their potential role in regulating protein function and activity [85, 86].

Understanding the proteins' secondary and tertiary structures and their potential antigenicity can provide crucial information about biologically relevant epitopes [87]. Secondary structure analysis indicated that random coils were the most prevalent structural feature at 58.52%, an important observation given that antibodies preferentially recognize these regions. Alpha helices, which contribute to protein stability, were also present [88]. Homology modeling using SWISS-MODEL, followed by refinement with GalaxyRefine, confirmed the structural reliability of TgAMA1, with a quality score of -6.12, indicating satisfactory overall structural integrity.

The host detects and targets the parasite through immunosurveillance, which involves the engagement and coordinated activation of innate and adaptive immune responses. Following *T. gondii* infection, dendritic cells (DCs), neutrophils, and macrophages (MQs) rapidly migrate to the infection site as the first line of defense. These immune cells recognize the parasite mainly through Toll-like receptors (TLRs), triggering the release IL-12, a Th1 type cytokine, which activates CD4^+^ and CD8^+^ T cells. Additionally, IFN-*γ*, another key Th1-type cytokine, is released by natural killer (NK) cells. This immune response helps restrict parasite replication and limit its spread within the host. To further drive a specific humoral response, IL-4, a Th2-type cytokine, promotes B cell proliferation and differentiation [89–91]. In the present study, specific linear B cell epitopes, including HTL (CD4^+^) and CTL (CD8^+^), were predicted using various web-based tools. The CTL epitopes were identified as restricted to the A3, A2, and B7 supertypes of MHC I molecules, ensuring broad coverage across diverse human HLA populations.

According to supertype ranking, the epitopes “RGYRFGVWK,“ “LVADCTIFA,” and “TPPTPETAL” had the highest immunogenicity scores. Among the predicted HTL epitopes, “AVSYTAAGSLSEETP” was identified as the most antigenic peptide. These epitopes were further assessed for their ability to induce IFN-*γ* and IL-4 production. While CD8^+^ T cells release IFN-*γ* to limit *T. gondii* infection, B cell-mediated antibody production also plays a crucial role in host for protection against *T. gondii* infection [92, 93]. To identify highly immunogenic, nonallergenic, and soluble linear B cell epitopes, a multistep predictive approach was applied. This analysis led to the discovery of “CSVKGEPPDLTWYCFK” from ABCpred (VaxiJen score: 1.1396) and “ELLEKNSNIKASTDLG” characterizing immunogenic properties and identifying novel antigenic peptides before proceeding with in vitro and in vivo studies for vaccine development and anti-*T. gondii* drug discovery.

## 5. Conclusion

In this study, we utilized immunoinformatics tools to identify the potential immunogenic and antigenic epitopes of the TgAMA1 protein. Our analysis identified many promising potential AMA1 epitopes and showed that the protein is highly immunogenic and nonallergenic. These findings are crucial for vaccine development and anti-*T. gondii* drug discovery to control toxoplasmosis.

## Figures and Tables

**Table 1:** Links to all bioinformatics online servers used in the research.

**Online site**	**Function**	**Link**	**Setting**	**References**
ABCpred	Prediction of B cell epitope(s) in an antigen sequence, using artificial neural network	http://crdd.osdd.net/raghava/abcpred/	Threshold: 0.9 Epitope length: 16 Overlapping filter: ON	[35]
AlgPred	Prediction of protein allergenicity	https://webs.iiitd.edu.in/raghava/algpred/submission.html	Hybrid approach (SVMc + IgE epitope + ARPs BLAST + MAST)	[36]
AllergenFP 1.0	Prediction of protein allergenicity	https://www.ddg-pharmfac.net/AllergenFP/	Default	[37]
C-ImmSim	In silico immune simulation	http://kraken.iac.rm.cnr.it/C-IMMSIM/	Simulation volume: 10 Random seed: 12345 Simulation steps: 1050 Three injections at 4-week intervals (LPS-free) with time series of 1, 84, and 168 Number Ag to inject: 1000 Host HLA selection: default	[38]
ElliPro	Prediction of linear and discontinuous antibody epitopes based on a protein antigen's 3D structure	http://tools.iedb.org/ellipro/	Minimum score: 0.5 Maximum distance (angstrom): 6	[39]
ExPASy ProtParam	Prediction of physicochemical parameters	https://web.expasy.org/protparam/	Default	[40]
GalaxyRefine	3D model refinement	http://galaxy.seoklab.org/cgi-bin/submit.cgi?type=REFINE	Default	[41, 42]
GOR IV	Protein secondary structure prediction	https://npsa-prabi.ibcp.fr/cgi-bin/npsa_automat.pl?page=npsa_gor4.html	Default	[43]
GPS-PAIL 2.0	Prediction of acetylation on internal lysines	http://pail.biocuckoo.org/	Default	[44]
IEDB	Antibody epitope prediction from protein sequences	http://tools.iedb.org/bcell/	Default	[45–50]
IEDB	Class I immunogenicity	http://tools.iedb.org/immunogenicity/	Default	[51]
IEDB MHCII	MHC II binding predictions	http://tools.iedb.org/mhcii/	Default	[52]
IFNepitope	A server for predicting and designing interferon-gamma–inducing epitopes	http://crdd.osdd.net/raghava/ifnepitope/	Default	[53]
IL4-pred	In silico platform for designing and discovering of interleukin-4-inducing peptides	http://crdd.osdd.net/raghava/il4pred/	Default	[54]
NetCTL 1.2	Prediction of CTL epitopes in protein sequences	https://services.healthtech.dtu.dk/services/NetCTL-1.2/	Threshold: 0.75	[55]
NetNGlyc 1.0	Predicts N-glycosylation sites	https://services.healthtech.dtu.dk/services/NetNGlyc-1.0/	Default	[56]
NetOGlyc 4.0	O-GalNAc (mucin type) glycosylation sites in mammalian proteins	https://services.healthtech.dtu.dk/services/NetOGlyc-4.0/	Default	[57]
NetPhos 3.1	Prediction of phosphorylation sites in eukaryotic proteins	https://services.healthtech.dtu.dk/services/NetPhos-3.1/	Default	[58]
PepCalc	Peptide property calculator	https://pepcalc.com/	Default	[59]
ProSA-web	Prediction of the model's overall quality	https://prosa.services.came.sbg.ac.at/prosa.php	Default	[60]
Protein-Sol	Protein solubility	https://protein-sol.manchester.ac.uk/	Default	[61]
PSORT II	Prediction of subcellular localization(s)	http://psort.hgc.jp/form2.html	Default	[62, 63]
Structure assessment tool	Prediction of quality and structural features of crude and refined models	https://swissmodel.expasy.org/assess	Default	[64]
SVMTriP	A tool to predict linear antigenic B cell epitopes	http://sysbio.unl.edu/SVMTriP/	Epitope length: 16	[65]
SWISS-MODEL	Protein structure homology modeling	https://swissmodel.expasy.org/	Default	[66]
TMHMM ver. 2.0	Prediction of transmembrane helices in proteins	https://services.healthtech.dtu.dk/services/TMHMM-2.0/	Default	[67, 68]
ToxoDB	TgAMA1 protein sequence retrieval	https://toxodb.org/toxo/app	N/A	[34]
VaxiJen v. 2.0	Protein antigenicity	http://www.ddg-pharmfac.net/vaxijen/VaxiJen/VaxiJen.html	Target organism: parasite (threshold: 0.5)	[69]

**Table 2:** The physicochemical properties of the TgAMA1 protein.

**Characteristics**	**Finding**	**Remarks**
Number of amino acids	569	Suitable
Molecular weight (MW)	63,020.59	High
Theoretical pI	5.78	Acidic
Chemical formula	C_2762_H_4254_N_768_O_865_S_30_	—
Extinction coefficient (at 280 nm in H_2_0)	77,905	—
Total number of negatively charged residues (Asp + Glu)	69	—
Total number of positively charged residues (Arg + Lys)	57	—
Estimated half-life (mammalian reticulocytes, in vitro)	30 h	—
Estimated half-life (yeast, in vivo)	> 20 h	—
Estimated half-life (*E. coli*, in vivo)	> 10 h	—
Instability index (II)	44	Unstable
Aliphatic index	64.62	—
Grand average of hydropathicity (GRAVY)	−0.531	Hydrophilic

## Data Availability

The data that support the findings of this study are available from the corresponding authors upon reasonable request.

## Supporting Information

Supporting Information Additional supporting information can be found online in the Supporting Information section. Table S1. Epitope prediction for the TgAMA1 protein specific to CTL is limited to three MHC supertypes (A2, A3, and B7). Table S2. Prediction of the HTL epitope for the TgAMA1 protein via the entire HLA reference set available on the IEDB server and subsequent screening regarding antigenicity, IFN-*γ* induction, and IL-4 induction. Table S3. Final screening of the common continuous B cell epitopes of the TgAMA1 protein predicted using the ABCpred and SVMTriP web servers. Table S4. Summary of conformational B cell epitopes of the TgAMA1 protein predicted by ElliPro.
