# Supplementary material for: In Silico–Based Investigation of the Immunogenicity and Biochemical Attributes of Toxoplasma gondii Apical Membrane Antigen 1 (TgAMA1)
Source: J Parasitol Res. 2025 Apr 12;2025:3514414. doi: 10.1155/japr/3514414 (PMC12009177; doi:10.1155/japr/3514414)
Supplement: Supporting Information — Additional supporting information can be found online in the Supporting Information section. Table S1. Epitope prediction for the TgAMA1 protein specific to CTL is limited to three MHC supertypes (A2, A3, and B7). Table S2. Prediction of the HTL epitope for the TgAMA1 protein via the entire HLA reference set available on the IEDB server and subsequent screening regarding antigenicity, IFN-γ induction, and IL-4 induction. Table S3. Final screening of the common continuous B cell epitopes of the TgAMA1 protein predicted using the ABCpred and SVMTriP web servers. Table S4. Summary of conformational B cell epitopes of the TgAMA1 protein predicted by ElliPro. [file 3514414.f1.docx]

**Supplementary Table 1.** Epitope prediction for the TgAMA1 protein specific to CTL is limited to three MHC supertypes (A2, A3, and B7).

| Supertype | Rank | Sequence | Score | Immunogenicity |
| --- | --- | --- | --- | --- |
| A2 Supertype | 1 | FIIPSNPSV | 1.4194 | -0.2411 |
|  | 2 | HLIYGSAYV | 1.1612 | -0.0523 |
|  | 3 | LVADCTIFA | 1.0980 | 0.1997 |
|  | 4 | GVASLLVLV | 1.0399 | -0.1466 |
| A3 Supertype | 1 | RGYRFGVWK | 1.2287 | 0.3610 |
|  | 2 | IMGGLRSLR | 1.1972 | -0.0630 |
|  | 3 | GLSSSTRSR | 1.1696 | -0.3977 |
|  | 4 | RSLRAARPY | 1.0688 | 0.1606 |
| B7 Supertype | 1 | RPPYRNNFL | 1.6262 | 0.0999 |
|  | 2 | TPPTPETAL | 1.2916 | 0.1745 |
|  | 3 | SASTSGNPF | 1.2354 | -0.1557 |
|  | 4 | YPLTSQASW | 1.2189 | -0.2983 |

Supplementary Table 2. Prediction of the HTL epitope for the TgAMA1 protein via the entire HLA reference set available on the IEDB server and subsequent screening regarding antigenicity, IFN-γ induction, and IL-4 induction.

| Allele | HTL epitope | Method | Percentile  rank | Antigenicity | IFN-γ inducing | | IL-4 inducing | |
| --- | --- | --- | --- | --- | --- | --- | --- | --- |
|  |  |  |  |  | **Result** | **Score** | **Result** | **SVM**  **score** |
| HLA-DRB1*04:01 | FKTVAMDKNNKATKY | Consensus (smm/nn/  sturniolo) | 0.01 | 0.8144 (Probable antigen) | Positive | 5 | Positive | 1.28 |
| HLA-DRB1*04:05 | RNYGFYYVDTTGEGK | Consensus (smm/nn/  sturniolo) | 0.02 | -0.1111 (Probable non-antigen) | Positive | 4 | Positive | 0.29 |
| HLA-DRB1*09:01 | AVSYTAAGSLSEETP | Consensus (smm/nn/  sturniolo) | 0.03 | 1.0580 (Probable antigen) | Negative | -0.7014 | Positive | 0.26 |
| HLA-DRB4*01:01 | GKHIELQQPDRPPYR | Consensus (smm/nn/  sturniolo) | 0.04 | 0.6116 (Probable antigen) | Positive | 0.2647 | Positive | 1.11 |
| HLA-DRB3*02:02 | PGGFNLNFVTPSGQR | Consensus (smm/nn/  sturniolo) | 0.07 | 0.4065 (Probable non-antigen) | Negative | -0.5053 | Positive | 0.24 |

Supplementary Table 3. Final screening of the common continuous B-cell epitopes of the TgAMA1 protein predicted using the ABCpred and SVMTriP web servers.

|  | Rank | Sequence | Score | VaxiJen  score | Allergenicity | Water  Solubility |
| --- | --- | --- | --- | --- | --- | --- |
| ABCpred | 1 | DQPHTYPLTSQASWND | 0.94 | 0.1007 | Negative | Poor |
|  | 2 | HIELQQPDRPPYRNNF | 0.94 | 0.5242 | Negative | Good |
|  | 3 | PPTPETALQCTADKFP | 0.91 | 0.2341 | Positive | Good |
|  | 4 | CWVKTFENDGVASDQP | 0.91 | 1.001 | Negative | Good |
|  | 5 | TDTVIERVESKAQCWV | 0.91 | 0.2237 | Negative | Good |
|  | 6 | AYVGENPDAFISKCPN | 0.91 | 1.1178 | Negative | Good |
|  | 7 | CSVKGEPPDLTWYCFK | 0.90 | 1.1396 | Negative | Good |
| SVMTriP | 1 | HMGLVGVASLLVLVAD | 1.00 | 0.3898 | Negative | Poor |
|  | 2 | DSKKRLCHILYVSMQL | 0.95 | 0.3454 | Negative | Good |
|  | 3 | ELLEKNSNIKASTDLG | 0.93 | 0.9435 | Negative | Good |
|  | 4 | GCYFAKRLDRNKGVQA | 0.83 | 0.5864 | Negative | Good |

**Supplementary Table** 4**.** Summary of conformational B-cell epitopes of the TgAMA1 protein predicted by ElliPro. server.

| No | Residues | No. of residues | Score |
| --- | --- | --- | --- |
| 1 | A:M1, A:I2, A:C3, A:S4, A:I5, A:M6, A:G7, A:G8, A:L9, A:R10, A:S11, A:L12, A:R13, A:A14, A:A15, A:R16, A:P17, A:Y18, A:S19, A:H20, A:Q21, A:S22, A:N23, A:T24, A:E25, A:T26, A:K27, A:H28, A:M29, A:G30, A:L31, A:V32, A:G33, A:V34, A:A35, A:S36, A:L37, A:L38, A:V39, A:L40, A:V41, A:A42, A:D43, A:C44, A:T45, A:I46, A:F47, A:A48, A:S49, A:G50, A:L51, A:S52, A:S53, A:S54, A:T55, A:R56, A:S57 | 57 | 0.927 |
| 2 | A:R512, A:N513, A:K514, A:G515, A:V516, A:Q517, A:A518, A:A519, A:H520 | 9 | 0.913 |
| 3 | A:K534, A:R535, A:P536, A:S537, A:D538, A:L539, A:M540, A:Q541, A:E542, A:A543, A:E544, A:P545, A:S546, A:F547, A:W548 | 15 | 0.851 |
| 4 | A:D549, A:E550, A:A551, A:E552 | 4 | 0.825 |
| 5 | A:R529, A:G530, A:A531, A:R532 | 4 | 0.825 |
| 6 | A:E524, A:Q526, A:S527, A:D528 | 4 | 0.784 |
| 7 | A:V449, A:C452, A:D470, A:C471, A:T472, A:A473, A:D474, A:E475, A:Q476, A:N477, A:E478, A:C479, A:G480, A:S481, A:N482, A:T483, A:A484, A:L485, A:I486, A:A487, A:G488, A:L489, A:A490, A:V491, A:G492, A:G493, A:V494, A:L495, A:L496, A:L497, A:A498, A:L499, A:L500, A:G501, A:G502, A:G503, A:C504 | 37 | 0.716 |
| 8 | A:E59, A:S60, A:Q61, A:T62, A:L63, A:S64, A:A65, A:S66, A:T67 | 9 | 0.663 |
| 9 | A:F174, A:P175, A:M176, A:E177, A:L178, A:L179, A:E180, A:K181, A:N182, A:S183, A:N184, A:I185, A:K186, A:A187, A:R224, A:E553, A:N554, A:I555, A:E556, A:Q557, A:D558, A:G559, A:E560, A:T561, A:H562 | 25 | 0.64 |
| 10 | A:Q434, A:C435, A:T436, A:A437, A:D438, A:K439, A:F440, A:P441, A:D442, A:C459, A:V460, A:G461, A:G462 | 13 | 0.609 |
| 11 | A:E104, A:V105, A:D106, A:G107, A:T108, A:Q127, A:Q128, A:P129, A:D130, A:R131, A:P132, A:P133, A:Y134, A:N136, A:N137, A:L139, A:E140, A:D141, A:V142, A:P143, A:T144, A:E145, A:K146, A:E147, A:Y148, A:K149, A:Q150, A:S151, A:G152, A:N153, A:P154, A:L155, A:P156, A:G157, A:G158, A:N160, A:L161, A:N162, A:F163, A:V164, A:T165, A:P166, A:S167, A:G168, A:Q169, A:R170, A:I171, A:S172, A:P173, A:V202, A:A203, A:M204, A:D205, A:K206, A:N207, A:N208, A:K209, A:A210, A:T211, A:K212, A:M236, A:E237, A:G238, A:K239, A:K240, A:Y241, A:C242, A:S243, A:V244, A:K245, A:G246, A:E247, A:P248, A:P249, A:D250, A:L251, A:T252, A:W253, A:Y254, A:C255, A:P343, A:L344, A:T345, A:S346, A:Q347, A:A348, A:S349, A:W350, A:N351, A:D352, A:W353, A:L356, A:V563, A:M564, A:V565, A:E566, A:G567, A:D568, A:Y569 | 99 | 0.606 |
| 12 | A:R317, A:D377, A:T378, A:T379, A:G380, A:E381 | 6 | 0.573 |
| 13 | A:S188, A:P419, A:S420, A:N421, A:P422, A:S423, A:V424 | 7 | 0.528 |
